# Smoking-associated black hairy tongue in a middle-aged male

**DOI:** 10.11604/pamj.2026.53.170.52141

**Published:** 2026-04-22

**Authors:** Janani Gopalakrishnan, Krishna Prasanth Baalann

**Affiliations:** 1 Department of Community Medicine, Sree Balaji Medical College and Hospital, Chromepet, Chennai, Tamil Nadu, India

**Keywords:** Black hairy tongue, hyperkeratosis, filiform papillae, burning mouth syndrome, oral hygiene, smoking

## Image in medicine

Black hairy tongue (lingua villosa nigra) is a harmless oral condition that happens when the filiform papillae on the back of the tongue grow longer and bigger because they do not shed keratin properly. Keratin builds up and forms hair-like projections that can trap food particles, bacteria, fungi, and tobacco residue. This can change the color of the hair from brown or black to yellow or green. A 54-year-old man had hair-like growth on his tongue for 20 days, along with a burning feeling for one week and a change in taste for five days. The patient had been smoking for 32 years, drinking alcohol on occasion, and not taking care of his teeth. An examination of the mouth showed long, black, and yellowish-green filiform papillae on the back and middle third of the tongue, but not on the tip. It was not possible to scrape off the lesion. The patient's history and clinical appearance led to the diagnosis of black hairy tongue. Management included brushing or scraping the tongue, using an antiseptic mouthwash, improving oral hygiene, quitting smoking, and changing the diet. During follow-up, the patient showed signs of improvement.

**Figure 1:**
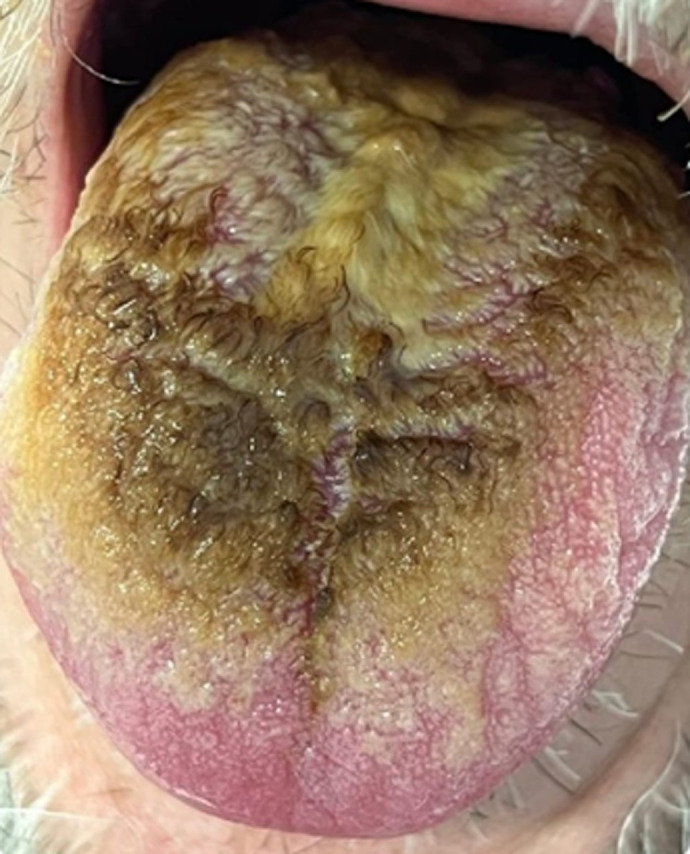
dorsal surface of the tongue showing yellowish-brown elongated filiform papillae with hair-like projections, consistent with black hairy tongue

